# Fluid-therapy for Brain Surgery: A Narrative Review

**DOI:** 10.4274/TJAR.2025.251489

**Published:** 2026-02-09

**Authors:** Maria Paola Lauretta, Luca Marino, Başak Akça, Boaz G. Samolsky Dekel, Federico Bilotta

**Affiliations:** 1 Policlinico Sant’Orsola-Malpighi, Department of Emergency-Urgency, Anaesthesia and Pain Therapy Unit, Bologna, Italy; 2 Sapienza University of Rome, Departments of Mechanical and Aerospace Engineering, Rome, Italy; 3 Hacettepe University Hospitals, Department of Anaesthesiology and Reanimation, Ankara, Türkiye; 4 University of Bologna, Department of Medical and Surgical Sciences, Bologna, Italy; 5 Sapienza University of Rome, Critical Care and Pain Medicine, Policlinico Umberto I, Department of Anaesthesiology, Rome, Italy

**Keywords:** Blood products, brain surgery, colloids, crystalloids

## Abstract

Brain surgery presents unique challenges to the anaesthesiology team in terms of complexity of patients and procedures. Managing fluid-therapy in this setting requires profound knowledge of different types of fluids and administration regimens. This review focuses on updated information about fluid therapy in elective and emergency brain surgery with specific insight on the clinical outcomes of patients.

**Main Points**

• Fluid-therapy in brain surgery affects the outcome.

• Specific hot points include: types of fluids and types of administration regimes.

• Fluid-therapy in neuro-anesthesia requires profound knowledge.

## Introduction

Fluid therapy (FT) in neurosurgical patients significantly impacts the early and long-term clinical course.^1, 2^ Perioperative fluid management in brain procedures should optimize cerebral perfusion and haemodynamic stability, (both essential to guaranteeing neuronal homeostasis), maintain an adequate circulating blood volume, preserve cerebral perfusion pressure (CPP), mean arterial pressure (MAP), and intracranial pressure (ICP), and minimize cerebral oedema.^3, 4^ Excessive fluid volumes can result in acute cardiac failure, pulmonary oedema, or cerebral oedema, while a disproportionate restriction may lead to hypotension.^5^ The clinical scenario is even more challenging in neurosurgery due to the use of osmotic diuretics, the long duration of surgeries, the major fluid shifts, the difficulty in assessing blood loss under the drapes, and the possibility of intraoperative central diabetes insipidus (CDI).^6, 7^ New clinical evidence related to periprocedural FT in neuro-anaesthesia makes it appropriate to summarize the most recent insights.

The aim of the present narrative review is to report clinical evidence related to periprocedural FT in patients undergoing brain surgery. FT can include crystalloids, colloids, blood-derived components, and several possible administration regimen infusion strategies: liberal, restrictive, goal-directed, etc.^8, 9^ Details on types of fluids and administration regimens, with specific insights on FT in elective and emergency brain surgery, will be provided in different sections. The supratentorial tumours and trans-sphenoidal surgery are considered paradigmatic of elective surgical procedures where FT plays a crucial role in clinical outcomes. Analogously, traumatic brain injury (TBI) and subarachnoid haemorrhage (aSAH) are discussed to review the impact of FT in emergency brain surgery.

### Types of Fluids and Administration Regimes in Brain Surgery

Perioperatively, the principal aim of fluid administration is to restore and maintain intravascular volume, organ perfusion, and ultimately, substrate delivery such as oxygen, electrolytes, and glucose.^10^ Furthermore, FT influences metabolite clearance, power of hydrogen homeostasis, medication supply, temperature control, and coagulation control.^11^ Among different fluid types, the use of crystalloids, colloids, and blood products deserves discussion.

Crystalloid is the term commonly applied to solutions that contain water and low molecular weight (MW) <500 g mol^-1^, solutes that may be charged (e.g. Na^+^, Cl^-^, Mg^2+^, K^+^) or uncharged (e.g. glucose or mannitol). Crystalloids are inexpensive, easy to store, with a long shelf life; they are readily available in a variety of formulations, require no special compatibility testing, and have a very low incidence of adverse reactions, with no religious objections to their use.^12^ Crystalloids are categorized as hyperosmolar solutions by the inclusion of electrolytes [e.g., Na^+ ^and Cl^-^, as in 3% hypertonic saline (3% HTS)] or low MW solutes, such as mannitol (MW 182) or glucose (MW 180). Hyperosmolar crystalloids (mannitol and HTS), in the presence of a normal blood-brain barrier (BBB), increase the osmotic gradient between the intravascular and cellular/interstitial compartments, leading to reductions in brain water content, brain volume, and ICP.^13^

Hypo-osmolar crystalloids [0.45% normal saline (0.45% NS) or dextrose 5% in water], when given in large amounts to neurosurgical patients, reduce plasma osmolality, drive water across the BBB, and increase cerebral water content, and ICP. Therefore, they should be avoided except in cases of CDI.^14^ Iso-osmolar crystalloids such as 0.9% NS and Plasma-Lyte have an osmolarity of approximately 300 mOsm L^-1^. These solutions neither change plasma osmolarity nor increase brain water content. However, caution should be exercised regarding large volumes of Ringer’s lactate (RL) with an osmolarity ≈273 mOsm L^-1^. Plasma-Lyte and the RL solutions contain bicarbonate precursors. These anions (e.g., lactate) are the conjugate base of the corresponding acid (e.g., lactic acid) and do not contribute to the development of acidosis, as they are administered with Na^+^ rather than hydrogen. The metabolism of lactate in the liver results in the production of an equivalent amount of bicarbonate.^15^

Colloids are solutions with a MW above 30,000 g mol^-1^ and have an oncotic pressure similar to that of plasma (Table 1). Colloid solutions are categorized according to the naturally occurring human plasma derivatives (5% and 25% albumin solutions, plasma protein fraction, fresh frozen plasma, and immunoglobulin solutions) and semisynthetic colloids [gelatins, dextrans (MW 40 and 70), and hydroxyethyl starch (HESs)].^16^ Colloids remain in the intravascular space for longer, and are used for volume expansion and to sustain blood pressure (BP) without associated complications from fluid overload. During active bleeding, more than 90% of the infused iso-oncotic colloids remain in the intravascular compartment. Possible negative effects, such as renal injury and coagulopathy, should be considered, and colloids should be used with caution in neurosurgical patients, in line with the do no harm principle. It is reasonable, therefore, to infuse colloids not before but when relative hypovolemia occurs. If a considerable amount of blood has been lost, replacement with blood products may be appropriate; however, increased organ dysfunction and poorer clinical outcomes are associated with “liberal” red blood cell (RBC) transfusion strategies. A “restrictive” strategy, intended to maintain haemoglobin at 7 to 9 g dL^-1^, along with a normovolemic anaemia, reduces morbidity and the risk of vasospasm after aSAH.^3, 17^ In conclusion, in patients undergoing neurosurgery, hypotonic solutions such as RL should be avoided to minimize cerebral fluid accumulation. Colloids and RBCs should be used with caution, tolerating normovolemic anemia.^3, 13, 14, 15, 16, 17^

Administration regimes (the amount and modality of administered fluids) also deserve a dedicated discussion; these include liberal, restrictive, goal-directed, and goal-directed haemodynamic FT. Several randomized controlled studies have compared restricted with liberal in patients undergoing major surgeries.^18, 19, 20^ In a randomized assessor-blinded multi-centre trial held in 2003, two perioperative fluid regimens were compared.^18^ Patients in the “liberal” group gained body weight and had more perioperative complications than the “restrictive” group. In that study, other evidence demonstrated that patients in the “restrictive” group had increased rates of surgical site infection and high risks of acute kidney injury.^19^ Goal-directed fluid therapy (GDFT) is a fluid regimen that optimizes predefined targets based on directly measured haemodynamic parameters, such as cardiac output (CO), the cardiac index (CI), stroke volume (SV), stroke volume variation (SVV), pulse pressure variation (PPV), systolic pressure variation, and the pleth variability index (PVI).^20^ SVV is a sensitive predictor of fluid responsiveness during brain surgery.^21^ After the induction of anaesthesia and before the start of the surgical procedure, SVV more sensitively predicts an increase above 10% in SV compared to MAP, CO, heart rate (HR), or central venous pressure (CVP). In a comparison of two GDFT regimens (with threshold SVV values set at 10 for the “low SVV” group and at 18 for the “high SVV” group) for supratentorial tumour resection, the low SVV group had lower postoperative serum lactate levels, a shorter ICU stay, and a lower incidence of postoperative neurologic events.^22^ PPV and PVI have also been reported to be good predictors of fluid reactivity during brain surgery.^23, 24, 25, 26, 27, 28, 29, 30, 31, 32, 33, 34, 35^ According to a recent trial held in India in patients undergoing brain tumour surgery in supine position, between a “CVP group,” which maintained a CVP of 5-10 cm H_2_O, and a “PPV group,” which maintained a PPV below 13%, the latter had better postoperative haemodynamic stability and less postoperative fluid requirements.^23^

In a trial randomizing patients with supratentorial tumour resection, “PPV-guided” GDFT exhibited better haemodynamic stability, brain condition, and organ perfusion than the “standard care” group targeting a CVP over 8 cm H_2_O.^24^ Another trial held in 2023 analyzed 74 patients undergoing neurosurgery for supratentorial mass and compared the efficacy of PVI versus PPV in guiding GDFT.^25^ Both groups received a baseline 2 mL kg^-1^ h^-1^ RL infusion, and additional fluid boluses of 250 mL of colloid if PVI >15% or PPV >13% for at least five minutes. The PVI- and PPV-guided GDFT strategies showed no significant difference in postoperative lactate values, with a *P* value of 0.18. Similarly, the mean total fluid administered, mean blood loss, length of ICU stay, and emetic and hypotension episodes showed no significant differences between the groups. More recently, an approach called "GDHT", guided by an algorithm based on non-invasive haemodynamic monitoring, has been investigated in major surgery.^26^ The aim is to optimize haemodynamic parameters such as CO and CI, which are essential for oxygen delivery to tissues, and for organ perfusion.

The positive effects of GDHT on patient-oriented outcomes were demonstrated in neurosurgery by a single-centre randomized pilot study with an enrolment target of 34 adult patients scheduled for elective cerebral procedures.^27^ The authors randomly assigned the patients to “control” and “GDHT” groups. The control group received standard therapy during surgery and aimed for a MAP above 65 mmHg, whereas the GDHT group received FT guided by an algorithm based on non-invasive haemodynamic monitoring. Specifically, after the determination of an optimal CI above 2.5 L·min·m^2^, the authors aimed to maintain SVV below 15%. The GDHT protocol was safe, and no patients in either group required therapy during surgery or 24 h after surgery, for unsatisfactory brain tissue relaxation or brain oedema. Major complications occurred in two patients in the GDHT group and six patients in the control group. A larger randomized trial evaluating the effects of GDHT on the incidence of postoperative complications in elective neurosurgery should be safe and feasible. 

In conclusion, while considering the administration regimens of FT, the parameters CI, SVV, PPV, and PVI seem to appropriately guide GDFT and GDHT, offering better tissue perfusion and lower perioperative complications than the standard of care, CVP or MAP-based approaches.^19, 20, 21, 22, 23, 24, 25, 26, 27^

### Fluid Therapy in Elective Brain Surgery

**Supratentorial Brain Tumours:** In elective oncological procedures for supratentorial tumours, hypotonic solutions, such as the LR solution, should be avoided to minimize cerebral fluid accumulation.^15^ In contrast, 0.9% NS, an isotonic crystalloid, is widely used because it is thought to reduce the risk of cerebral oedema.^28^ However, since 0.9% NS has equal amounts of sodium and chloride (154 mEq L^-1^), hyperchloremic metabolic acidosis may occur when a large amount is administered, as its chloride concentration is higher than the normal plasma chloride concentration (96-106 mEq L^-1^). Based on the above, an isotonic balanced solution, such as Plasma-Lyte A, is preferred over 0.9% NS in neurosurgical oncological patients because of the lower risk of metabolic acidosis and renal injury.^28, 29^

Intraoperatively, cerebral protection (related to the tumour debulking and dura incision) is provided with different strategies intended to reduce the impact and duration of high ICP: osmotherapy with either mannitol, a non-metabolized alcohol derivative of mannose, or 3% HTS, is the recommended first-line medical intervention to optimize cerebral perfusion through brain relaxation, thereby preventing neurological deterioration.^30^ In a prospective randomized study, 74 patients with American Society of Anesthesiologists (ASA) I to III scheduled for intracranial tumour surgery were enrolled to compare the effects of equi-volume, equi-osmolar solutions of mannitol and HTS on brain relaxation and postoperative complications.^31^ Patients received a 3.75 mL kg^-1^ intravenous infusion of either 3.2% HTS (n = 36) or 20% mannitol (n = 38). The surgeon assessed the condition of the brain using a 4-point scale after opening the dura. Patients who were administered 3.2% HTS had more brain relaxation compared with those who received mannitol (*P *<0.05). There were no significant differences between the groups in postoperative complications or in the length of ICU or hospital stay. The results suggest that HTS may provide better brain relaxation than mannitol during elective intracranial surgery for a tumour.

Preoperative anaemia management, such as with iron-deficiency correction or blood conservation strategies, is crucial in patients undergoing elective cerebral resection. In a recent retrospective analysis of patients who underwent primary glioblastoma resection between September 2009 and October 2019, complication rates were significantly higher among patients who received RBC transfusions than among those who did not-pneumonia (*P *<0.0001), sepsis (*P*=0.0013), pulmonary embolism (*P*=0.0061), and seizures (*P* <0.0001) - highlighting the importance of minimizing preoperative anaemia and intraoperative blood loss in elective neurosurgery.^17^

**Infratentorial Surgery: **Trans-sphenoidal surgery near the neurohypophysis for pituitary and sellar lesions can lead to salt and water disorders. Both CDI, a condition related to compromised arginine vasopressin synthesis that leads to hypernatremia and polyuria and was recently renamed arginine vasopressin deficiency, and the syndrome of inappropriate antidiuresis (SIAD), which leads to hyponatremia, may occur.^32^ Intraoperatively, in case of CDI, if the patient presents persistent increased urine output, hypo-osmolar crystalloids can be administered to correct hypernatremia (serum Na^+^ concentration >145 mmol L^-1^) and large volumes of dilute urine (osmolarity <250 mmol kg^-1^).^9, 10^ Also, in cases that warrant extended operative periods, pharmacological treatments for CDI can be considered, including vasopressin and analogues of vasopressin such as desmopressin (active on the same vasopressin receptors with a longer context-sensitive half-life), and hypo-osmolar crystalloids need to be discontinued to avoid subsequent hyponatremia.^14, 33, 34, 35^ In case of acute SIAD, if serum Na^+^ concentration falls below 135 and large volumes of dilute urine are eliminated (osmolarity <110 mmol kg^-1^), restrictive fluid regimes should be considered aiming to administer NS 1000 mL in 24 h (approximately 500 mL in theatre), and administration of 3% HTS in severely symptomatic cases, if presenting a serum Na^+^ concentration <120 mmol L^-1^, is recommended.^35^

### Fluid Therapy in Emergency Brain Surgery

**TBI:** After trauma, when the BBB is mechanically damaged and the cerebral inflammatory response is activated, initial rapid infusion of large volumes of mannitol, and a hypertonic crystalloid solution is the current standard of care for people with combined haemorrhagic shock and TBI to restore BP and blood volume.^36^ This approach is especially helpful in preventing subsequent ischemic brain damage by aiming for normovolemia and a haematocrit above 30%.^37^ However, the role of colloids needs to be clarified. The Saline versus Albumin Fluid Evaluation (SAFE) study randomized critically ill patients to receive either 4% albumin or NS fluid resuscitation over 28 days.^38^ Although there was no overall difference in 28-day mortality between the groups, there was a trend towards increased mortality in patients with trauma, randomized to albumin resuscitation. This increased mortality appeared to be driven by trauma patients with TBI compared with those with trauma without TBI. A post-hoc analysis of patients with TBI randomized in the SAFE trial confirmed that resuscitation with albumin, as compared with 0.9% NS, was associated with increased mortality at 24 months, as elevated albumin extravasation in the brain worsened cerebral oedema and increased interstitial oncotic pressure.^39^

Among plasma expanders, the role of gelatin and HES is extensively explored in the literature.^40, 41^ In several trials, haemodilution with gelatine and HES significantly impaired clot formation compared to crystalloid solutions; additionally, extravasation in the brain made cerebral oedema worse. Therefore, FT with crystalloid is more effective than FT with colloid in patients with TBI.^42^ When osmotherapy with either mannitol or HTS is recommended to optimize CPP, a water shift from intracellular to extracellular (and thus intravascular) compartments is facilitated, leading to CO augmentation.^43, 44^ Moreover, HTS can directly improve myocardial performance through a reduction in myocyte oedema and an increase in myocardial uptake of Ca^2+^.^45^ Although available data suggest that both mannitol and HTS promote an augmentation of CO, this effect seems to be more pronounced after HTS than after mannitol administration.^46^ Furthermore, mannitol increases diuresis, while HTS causes increases plasmatic Na^+^ concentration. These effects might be responsible, in part, for the overall therapeutic effects associated with osmotic therapies.^47^

**Endovascular procedures for aSAH: **Maintenance of normovolemia, haemodilution (haematocrit at 30% to 35%), and cerebral perfusion are essential to avoid vasospasm after the procedure, and hypotonic solutions such as RL are usually avoided intraoperatively.^9^ In a randomized controlled study held in Canada, 60% of enrolled patients with aSAH were dehydrated at the start of the endovascular coiling procedure.^48^ The authors randomized patients to receive either standard liberal FT or GDFT; better haemodynamic optimization was observed in the second group. In another trial, the authors randomized patients undergoing a clipping procedure for aSAH to receive either NS or a balanced salt solution, namely, Plasma-Lyte A. The second group exhibited a better renal and acid-base profile (lower base deficit and higher bicarbonate levels).^49^ According to a retrospective analysis on 54 patients with aSAH, mannitol or HTS is pivotal to ensure appropriate CPP when ICP spikes over 20 mmHg, especially during dura incision.^50^ The authors recommended “CPP-guided FT” aiming for CPP above 70 mmHg through optimization of both ICP and MAP. If hyponatremia occurs from the release of atrial natriuretic factor (cerebral salt wasting), treatment includes hydration with either normal or HTS to improve CPP.^51^

## Discussion

This narrative review demonstrates the impact of FT on neuro-anaesthesia in brain surgery. Crucial features of FT related to clinical outcomes have been reviewed and discussed.^1, 2^ In elective brain surgery, hypotonic solutions, such as RL, should be avoided to minimize cerebral fluid accumulation, except in infratentorial surgery if intraoperative CDI occurs.^35, 36^An isotonic balanced solution such as Plasma-Lyte A is the best option for intraoperative maintenance in standard conditions.^47^ In emergency brain surgery, after trauma or SAH, normovolemic anaemia should be tolerated; rapid infusion of large volumes of mannitol, and hypertonic crystalloid solution is the recommended first-line medical intervention to restore circulating volume.^48, 49, 50, 51^ Osmotherapy also optimizes cerebral perfusion through brain relaxation.^3^

A recent study reported the significant clinical impact of intra-operative over-hydration or excessive restriction on haemodynamic stability, serum lactate levels, urine output, and fluid retention. The study supported GDFT as the tailored optimal FT, guided by SVV, PPV, and PVI, rather than by CVP, MAP, HR, or inferior vena cava diameter.^19^

A limitation of the present review is the lack of a systematic methodology in the literature search; it may therefore be affected by uncertainty in study selection, which can potentially lead to bias. However, it presents some interesting and original insights. GDHT, based on non-invasive advanced haemodynamic monitoring, appears to be a promising approach in elective brain surgery. Specifically, after achieving a CI target above L·min·m^2^, maintaining SVV below 15% helps preserve an optimal CI. Furthermore, the GDHT protocol is safe and effective, even though further randomized trials evaluating its role in elective neurosurgery should be conducted.^26, 27^

## Conclusion

While numerous studies on intraoperative FT in brain surgery have been performed, evidence is too scarce to draw definitive conclusions regarding specific transfusion thresholds, and more trials exploring the GDHT regimen are necessary.

## Figures and Tables

**Table 1. Fluid Characteristics: Crystalloids Versus Colloids:** 

Crystalloids	Hypo-osmolar	Iso-osmolar	Hyperosmolar	Plasma expansion
-	<280 mOsm L^-1^	280-300 mOsm L^-1^	>300 mOsm L^-1^	20%
Colloids	Low MW (<150,000 g mol^-1^)	Medium MW (>150,000 g mol^-1^)	High MW (>350,000 g mol^-1^)	80-200%
-	69 to 150 kDa	Approx 150-350 kDa	>350 kDa	-

MW, molecular weight; Da, dalton
